# Anticandidal Efficacy of Erythrosine with Nano-TiO _2_ and Blue LED-Mediated Photodynamic Therapy against *Candida albicans* Biofilms on Acrylic Resin: A Preliminary Study

**DOI:** 10.1055/s-0043-1768165

**Published:** 2023-04-27

**Authors:** Teerasak Damrongrungruang, Subin Puasiri, Vichakorn Vongtavatchai, Chatchai Saeng-on, Teeruch Petcharapiruch, Aroon Teerakapong, Angkhana Sangpanya

**Affiliations:** 1 Division of Oral Diagnosis, Department of Oral Biomedical Sciences, Faculty of Dentistry, Khon Kaen University, Khon Kaen, Thailand; 2 Melatonin Research Program, Khon Kaen University, Khon Kaen, Thailand; 3 Laser in Dentistry Research Group, Khon Kaen University, Khon Kaen, Thailand; 4 Division of Dental Public Health, Department of Preventive Dentistry, Faculty of Dentistry, Khon Kaen University, Khon Kaen, Thailand; 5 Division of Periodontology, Department of Oral Biomedical Sciences, Faculty of Dentistry, Khon Kaen University, Khon Kaen, Thailand

**Keywords:** *C. albicans*, erythrosine, light emitting diode, nano-titanium dioxide, nystatin, photodynamic therapy

## Abstract

**Objective** Incorporating an enhancer such as nano-titanium dioxide into antimicrobial photodynamic therapy can improve treatment outcome.

This study aimed to compare the anticandidal efficacy of photodynamic therapy by erythrosine with nano-titanium dioxide (nano-TiO _2_ ) stimulated by a blue light emitting diode with three standard dental antifungal agents.

**Materials and Methods** *Candida albicans* biofilms on acrylic resin plates were treated for 15 minutes with either nystatin, fluconazole, Polident, 220µM erythrosine + 1% (w/w) nano-TiO _2_ + 15 J/cm ^2^ blue light photodynamic therapy (Ery PDT), or distilled water. For the Ery PDT group, blue light was applied for 1 minute after incubation. After 1, 3, and 6 hours, the colony forming units in log10 (log _10_ CFU/mL) were compared. The ultrastructure of *C. albicans* on the acrylic resin plates treated with erythrosine + nano-TiO _2_ + blue light was examined using transmission electron microscopy at magnification of 30,000x.

**Results** After 1 hour, nystatin, Polident, and Ery PDT indifferently inhibited *C. albicans* . At 6 hours, Ery PDT reduced the number of viable *C. albicans* in biofilms by 0.28log _10_ CFU/mL, which was equal to the effect of fluconazole and Polident. Transmission electron microscopy demonstrated that Ery PDT altered the *C. albicans* cell morphology by inducing cell wall/membrane rupture.

**Conclusion** Photodynamic therapy with erythrosine + nano-TiO _2_ + blue light at low light power density (15 J/cm ^2^ ) was as effective at inhibiting *C. albicans* biofilm on acrylic resin as fluconazole and Polident.

## Introduction

Denture stomatitis is a condition caused primarily by fungal growth on all surfaces of dentures that are worn for prolonged times and/or following inadequate cleaning of dentures. *Candida albicans* is the most common fungal species causing denture stomatitis, and oral candidiasis has been reported in 24 to 60% of denture wearers. 1 There are several well-known contributing factors for oral candidiasis including the age of the denture wearer and the period of denture usage, hygiene habits, candidal load, host immune condition, nocturnal denture wear, occlusion, and denture retention and stability. The main symptoms of oral candidiasis can significantly reduce the quality of life. 2 The first line of treatment for denture stomatitis is an antifungal agent such as nystatin or miconazole; however, biofilm-associated *C. albicans* can be resistant to these agents. 3 To prevent microbial growth, dentures can be soaked in chemical agents such as chlorhexidine and/or sodium hypochlorite and cleaned with denture cleaners (for instance Polident). The key ingredient in Polident is sodium carbonate peroxide, an oxidizing agent that can kill algae and fungi. 4 However, the long-term use of denture cleaners can roughen denture surfaces and change the color of the acrylic. 5 6 Therefore, alternative denture cleansing aids that employ natural products or laser cleaning are recommended, especially for individuals with fragile oral mucosa. 7

*C. albicans* can cause many health problems including urinary tract infections and gastrointestinal inflammation, and emerging evidence suggests that *C. albicans* is associated with periodontal disease chronicity and severity. 8 Moreover, *C. albicans* infection may be capable of malignant transformation by triggering Th17-mediated inflammation. 9 Thus, to prevent serious health consequences from candidiasis, effective long-lasting treatments need to be developed.

Antimicrobial photodynamic therapy (aPDT) is a promising treatment that activates photosensitizers with specific light to inhibit/kill microorganisms. 10 One of its benefits is its prolonged antimicrobial effects. Hormdee et al demonstrated that a single application of antimicrobial PDT using *Curcuma longa* extract gel as the photosensitizer could maintain a reduction in the subgingival microflora for at least 6 weeks. 11 This is a significant improvement over traditional antimicrobial treatments, which often require repeated applications to maintain their effectiveness. We hypothesized that antimicrobial PDT could be as effective as antifungal drugs for denture disinfection without any associated changes to the denture surface properties. Common photosensitizers including porphyrin, phthalocyanine, and toluidine have all been proven to be effective in antimicrobial PDT applications, 12 but they are not capable of binding to the oral microbiota. Moreover, they can only absorb light in the red region, 12 which is not commonly available in dental clinic. One commonly used dental biofilm disclosing agent that also possesses photosensitizing ability is erythrosine (Ery). Previous studies have reported that Ery activated by a tungsten light could kill *Porphyromonas gingivalis* 13 and 220-µM Ery activated with a blue dental LED (250 mW/cm ^2^ ) at 15 J/cm ^2^ inhibited *C. albicans* biofilm growth. 14 Thus, we hypothesized that employing these specific light parameters might be a promising means for inhibiting *C. albicans* on acrylic denture bases, which could provide a new paradigm for denture cleanliness care protocols.

Employing combinations of photosensitizers in antimicrobial PDT is emerging as an effective way to increase antimicrobial activity over protocols using a single photosensitizer. 14 15 Among these, photocatalysts are among the most promising alternatives due to their ability to enhance the photodynamic reaction. 16 Nano-titanium dioxide (TiO _2_ ) consists of particles of TiO _2_ with an ultrafine crystalline structure and photocatalytic properties. 16 Nano-TiO _2_ was shown previously to increase the efficacy of blue light (BL) stimulated Ery killing of *C. albicans* biofilm, 14 but, to our knowledge, this has not been investigated on acrylic plates.

A light curing unit is a machine used to cure dental restorative materials. These units employ blue light emitting diode (LED)s to provide light at wavelengths of 400 to 500 nm. 17 A previous study successfully employed PDT to kill *C. albicans* on rat tongues using Photogem, a first-generation hematoporphyrin photosensitizer, activated by LEDs at wavelengths of 455 and 630 nm. 18 However, Photogem requires >30 minutes of exposure for activation due to its large molecular size. 19 20 Recently, antimicrobial PDT with Ery irradiated by a high energy density (63 J/cm ^2^ ) dental blue LED was performed with profound anticandidal effects. 21 But employing such a high level of energy might also have negative effects on normal oral cells. Therefore, developing novel treatment modalities that use common dental LEDs with photosensitizers with shorter incubation periods could provide more practical antimicrobial PDT.

Thus, we combined Ery and nano-TiO _2_ irradiated with a dental LED in PDT and examined its *C. albicans* inhibitory effects. We compared the antifungal biofilm effects of antifungal agents, a denture cleaning agent and PDT in an *in vitro* acrylic resin denture specimen setting. Additionally, we observed the ultrastructure of *C. albicans* cells on the resin acrylic surface after the PDT reaction to elucidate the morphological effects of our PDT regimen.

## Materials and Methods

### Study Design

This is an *in vitro* , laboratory-based study.

### Selection Criteria

We employed the commonly used dental acrylic resin and the dental blue LED light curing unit in our institute. The most common candidal species was selected as the independent variable.

### Acrylic Resin Specimen Preparation

Fabrication of 135 circular acrylic resin samples (diameter of 35 mm and thickness of 2 mm) was performed using a dental stone mold. Heat-processed acrylic resin (Meliodent) was mixed according to the manufacturer's recommendations and packed into a dental stone mold. Nine samples were then polymerized by a conventional heat method with metal flasks in an automatic polymerization water tank at 70°C for 8 hours, followed by 100°C for 1 hour. All samples were cooled overnight. Samples were then deflasked, and excess resin was sequentially removed. The acrylic resin surface was polished with Ecomet at 120 rpm for 4 minutes. Subsequently, all samples were immersed in distilled water at room temperature for over 50 hours to eliminate any residual monomers. Finally, all samples were put in Petri dishes, sealed by silicone sealers, and then sterilized with ethylene oxide gas.

### Preparation of *Candida albicans* Biofilms

*C. albicans* ATCC 10231 was cultured for 72 hours at 37°C in Sabouraud dextrose broth (Difco, Spark, MD, United States). It was adjusted to an optical density of 0.2 at 600 nm (10 ^7^ cells/mL) using a spectrophotometer (Beckman Coulter DU-730, Pasadena, CA, United States). *Candida* solution was subsequently incubated at 37°C on a 75 round/min shaking incubator for 90 minutes. Then, 3 mL of *C. albicans* suspension was pipetted into an acrylic plate in a Petri dish supplemented with 50 mM glucose in 1X yeast nitrogen base and incubated for 72 hours at 37°C in an incubator following the method of Thein et al 22 with medium renewed at 48 hours. Each Petri dish plate was then washed for 5 minutes with 3 mL of phosphate buffer saline (PBS) to remove loosely adhered cells before being tested for inhibition by antifungal agents or interventions.

### Antifungal Agent Preparation

Ery solution was prepared by dissolving 19.34 mg of Ery (Sigma-Aldrich, St. Louis, MO, United States) in 100 mL of deionized water. After sterilization with a 0.22-m pore syringe, 1.934 mg of nano-TiO _2_ powder (Sigma-Aldrich) was suspended at a concentration of 1% weight. The solution was kept in the dark until used. Denture cleanser was made by dissolving one denture cleanser tablet (Polident) in 200 mL of deionized water at room temperature. Fluconazole was prepared by dissolving 200 mg of fluconazole in a container of 20 mL of deionized water (final concentration 32 mM or 10 mg/mL). Nystatin (Tystatin) contained 100,000 USP Nystatin Units per milliliter. Distilled water in this study was a negative control.

### Light Source

The light source was a dental LED unit (BA OPTIMA 10). The Irradiation surface area was 0.28 cm ^2^ , the beam diameter was 6 mm, and the light-to-well bottom distance = 24 mm, 420 to 480 nm, power output of 1,200 mW (250 mW/cm ^2^ ) confirmed by an optical power meter model S210A (Thorlab Inc., Newton, NJ, United States), and 15 J/cm ^2^ .

### *Group Allocation* s

**Fig. 1:**
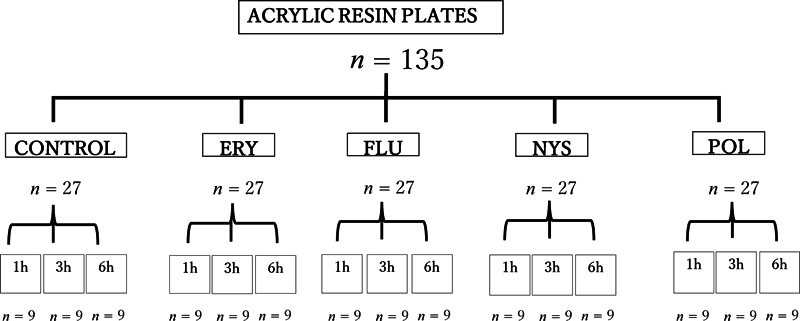
Group allocation (ERY, erythrosine; FLU, fluconazole; NYS, nystatin; POL, Polident).

Three milliliters of the antifungal agent sample were pipetted onto resin acrylic plate with biofilms ( Fig. 1 ). Ery with nano-TiO _2_ was soaked in the plate for 15 minutes and kept in a dark box. The Ery with nano-TiO _2_ groups were irradiated for 1 minute.

These agents were rinsed out three times with 3 mL PBS after 1, 3, and 6 hours. Two milliliters of PBS were pipetted into Petri dishes. The Petri plates were shaken for 15 minutes at 120 kHz using an ultrasonic machine (ultrasonic LEO-1502 ultrasonic cleaner, Taiwan) to disrupt and collect entire biofilms, and the suspensions were collected for cultivation. The suspensions were diluted to 1:1,000 and 10 µL were inoculated onto Sabouraud dextrose. After a 24-hour incubation period at 37°C, the number of CFU was counted (CFU/mL). The log _10_ base was used to convert all CFU/mL. All the experiments were performed in three replications.

Test group:

- 220-µM Ery + 1% TiO _2_ (w/w) + blue dental LED light.
- Fluconazole 1 capsule (200 mg) final concentration 32 mM or 10 mg/mL.
- Nystatin 100,000 U/mL.
- Polident 1 tablet (2.704 g); final concentration: 27.04 mg/mL.

Control group:

- Distilled water.

### Transmission Electron Microscope Analysis

A 1-mL suspension of control and Ery + nano-TiO _2_ groups were harvested, fixed with Karnovsky's solution at 4°C overnight (12 hours). The suspensions were washed four times for 15 minutes with PBS, followed by post-fixing for 30 minutes with 1% osmium tetroxide solution and washed twice for 15 minutes with PBS. The suspensions were dehydrated with graded ethanol, infiltrated with plastic solution (EPON 82000), and stiffened in a hot air oven at 60°C for 48 hours. The samples were cut with an ultramicrotome and observed by a transmission electron microscope (JEM-1010; JEOL Ltd, Tokyo, Japan) at magnification of 30,000x.

### Statistical Analysis

Descriptive analyses, median ± interquartile range, were used to evaluate log _10_ CFU/mL of each group. The normality test was performed using the Shapiro–Wilk test. The multiple comparisons of five groups at different times were evaluated using the Kruskal–Wallis test and Dunn–Bonferroni test; the significance level was set at *p* < 0.05. Intragroup repeated measurement comparison using mean ± SD at different time points was conducted with post hoc test by Bonferroni; the significance level was set at *p* < 0.05.

## Results

### Comparison of the Anticandidal Efficacy of PDT against *C. albicans* Biofilms

**Fig. 2:**
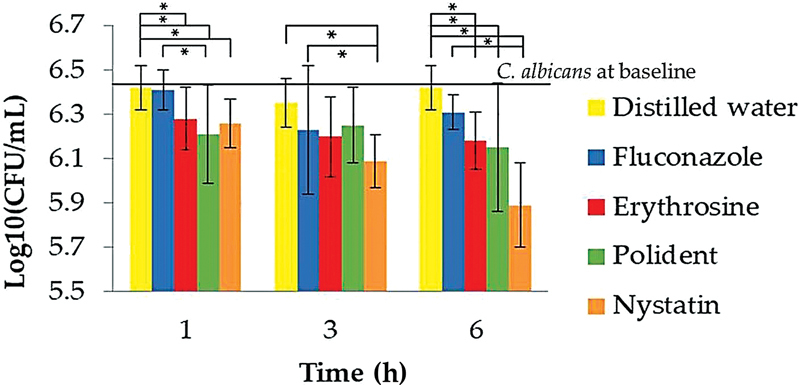
Comparison of the median log _10_ CFU/mL of *Candida albicans* by drop plate assay among various regimens at baseline 1, 3, and 6 hours (*= significant difference at *p* < 0.05). Negative control = distilled water; *n* = 9.

Fig. 2 presents the number of viable *C. albicans* recovered from biofilms following four treatments over 6 hours in log _10_ scale. At 1 hour, fluconazole did not reduce the *C. albicans* numbers compared with the controls. However, treatment with Ery PDT (220-µM Ery + 1% (w/w) TiO _2_ + BL) reduced the number of viable *C. albicans* by 30.87% to 6.28 ± 0.14 log _10_ CFU/mL ( *p* = 0.022 compared with the controls), Polident reduced *C. albicans* by 33.82% to 6.21 ± 0.22 log _10_ CFU/mL ( *p* = 0.017), and nystatin reduced *C. albicans* by 30.78% to 6.26 ± 0.11 log _10_ CFU/mL ( *p* = 0.003). In addition, Polident significantly reduced *C. albicans* numbers compared with fluconazole ( *p* = 0.028).

At 3 hours, fluconazole, Ery, and Polident reduced the number of viable *C. albicans* to 6.23 ± 0.23, 6.2 ± 0.18, 6.25 ± 0.17 log10CFU/mL, which corresponded to 19.84, 40.81, 39.02% reductions, respectively. But these reductions were not significant compared with the controls. Nystatin statistically significantly reduced the *C. albicans* numbers compared with the controls ( *p* < 0.001) and fluconazole ( *p* = 0.003).

At 6 hours, fluconazole did not reduce *C. albicans* numbers compared with the controls (6.15 ± 0.29 log _10_ CFU/mL, 25.91% reduction) but Ery, Polident, and nystatin all statistically significantly reduced *C. albican* s numbers compared with the controls. Ery reduced *C. albicans* numbers by 46.42% to 6.18 ± 0.13 log _10_ CFU/mL ( *p* = 0.007), Polident by 51.57% to 6.15 ± 0.29 log _10_ CFU/mL ( *p* = 0.002), and nystatin by 73.25% to 5.89 ± 0.19 log _10_ CFU/mL ( *p* < 0.001). In addition, nystatin reduced *C. albicans* numbers by more than fluconazole ( *p* = 0.001). Interestingly, 220µM Ery + 1% TiO _2_ + BL demonstrated no statistically significant difference in *C. albicans* reduction ability compared with nystatin ( *p* = 0.298).

### Intragroup Comparison of Anticandidiasis Activity at Different Time Points

**Fig. 3:**
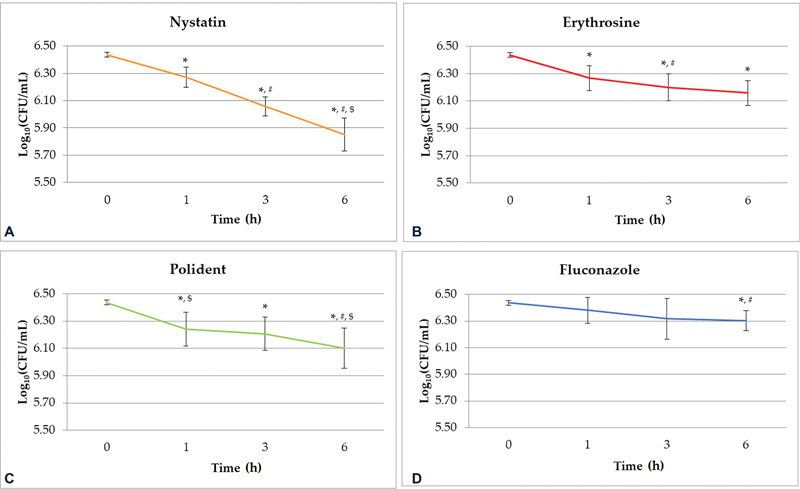
( **A** ) Nystatin 100,000 U/ml. ( **B** ) Erythrosine 220 μM +1%(w/w) Titanium dioxide and blue dental LED. ( **C** ) Polident 27.04 mg/ml. ( **D** ) Fluconazole 10 mg/ml. Comparison of mean log _10_ CFU/mL of viable *Candida albicans* determined by drop plate assay at 1, 3, and 6 hours. Control = zero hours, *n* = 9. *, #, $ = significant difference at *p* < 0.05 when compared with time 0, 1, and 3 hours, respectively. Changes in the number of *C. albicans* within each group among different time points were analyzed by pairwise comparison and Bonferroni's test.

Analysis of the number of viable *C. albicans* recovered from biofilms within treatment groups over time showed statistically significant different changes within each group ( Fig. 3 ).

Fig. 3A shows time-dependent inhibition of *C. albicans* by nystatin with statistically significant reductions in the number of viable *C. albicans* recovered from biofilms at 1 hour (6.27 ± 0.07 log _10_ CFU/mL 30.73%, *p* = 0.001), 3 hours (6.06 ± 0.07 log _10_ CFU/mL 57.85%, *p* < 0.001), and 6 hours (5.85 ± 0.12 log _10_ CFU/mL 73.23%, *p* < 0.001) compared with baseline at time zero (6.44 ± 0.02 log _10_ CFU/mL). Furthermore, comparison between times showed statistically significant reductions in *C. albicans* numbers from 1 to 3 hours ( *p* < 0.001), 1 to 6 hours ( *p* < 0.001), and 3 to 6 hours ( *p* = 0.025).

Ery PDT ( Fig. 3B ) also showed time-dependent inhibition of *C. albicans* , statistically significantly reducing the number of *C. albicans* at 1 hour (6.27 ± 0.09 log _10_ CFU/mL 30.81%, *p* = 0.005), 3 hours (6.20 ± 0.1 log _10_ CFU/mL 40.77%, *p* = 0.001), and 6 hours (6.16 ± 0.09 log _10_ CFU/mL 46.38%, *p* < 0.001), compared with baseline at time zero. In addition, there was a statistically significant reduction in the number of *C. albicans* from 1 to 3 hours ( *p* = 0.035).

Polident also showed time-dependent inhibition of *C. albicans* , statistically significantly reducing the number of *C. albicans* at 1 hour (6.24 ± 0.12 log _10_ CFU/mL 33.77%, *p* = 0.015), 3 hours (6.21 ± 0.12 log _10_ CFU/mL 38.98%, *p* = 0.03), and 6 hours (6.10 ± 0.15 log _10_ CFU/mL 51.54%, *p* = 0.001) compared with baseline at time zero ( Fig. 3C ). In addition, there were statistically significant reductions in the number of *C. albicans* at 6 hours compared with 1 hour ( *p* < 0.001) and 3 hours ( *p* = 0.004).

Fluconazole statistically significantly reduced the number of *C. albicans* recovered from biofilms at 6 hours (6.30 ± 0.08 log _10_ CFU/mL 25.85%) compared with the baseline at time zero ( *p* = 0.006) and 1 hour ( *p* = 0.001; Fig. 3D ).

### Ultrastructural Analysis of the Interaction between *C. albicans* and Erythrosine Nano-Titanium Dioxide by Transmission Electron Microscopy

**Fig. 4:**
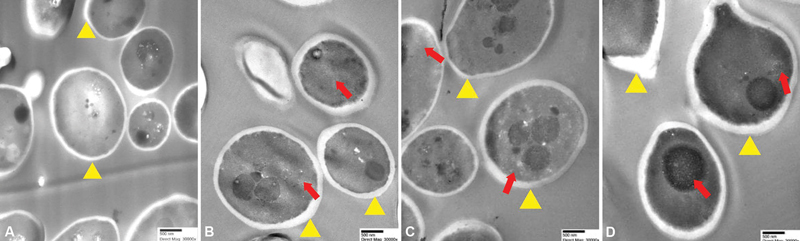
Transmission electron microscopy (TEM) photomicrographs of *Candida albicans* after exposure to erythrosine with nano-titanium dioxide for ( **A** ) untreated, ( **B** ) 1 hour, ( **C** ) 3 hours, and ( **D** ) 6 hours. *Arrowheads* indicate cell wall and *arrows* indicate nano-titanium dioxide particles (magnification of 30,000x; bar = 500 nm).

Fig. 4 presents representative transmission electron microscopy (TEM) micrographs (magnification 30,000x) of *C. albicans* cells treated with Ery + nano-TiO _2_ + blue LED. Fig. 4A shows an untreated *C. albicans* with well-conserved morphological features including a typically structured cell wall, cell membrane, and nucleus. After exposure to 220-µM Ery + 1% TiO _2_ + BL for 1, 3, and 6 hours ( Fig. 4B , C , and D , respectively), the *C. albicans* cells were generally enlarged, with thicker cell membranes and cell walls. The treated cells clearly displayed internalized and aggregated nanoparticles (NP; dimension <25 nm) that were distributed in the cytoplasm, submembrane, and nuclear envelope.

## Discussion

The present study demonstrated effective inhibition of *C. albicans* biofilm on acrylic resin denture base specimens by photodynamic therapy using 220-µM Ery + 1% (w/w) nano-TiO _2_ and blue dental LED at 15 J/cm ^2^ .

Notably, the efficacy of *C. albicans* inhibition by our PDT regimen in the present study was superior to fluconazole at 1 and 6 hours. Fluconazole is a first-line systemic drug with minimal side effects, 23 making it a potential candidate for use as a denture cleansing agent. However, fluconazole is not commonly used as denture soaking agent. The concept of using an antifungal agent to inhibit *Candida* on the surface of dentures in concordance with mucosal treatment to achieve maximal and fungicidal effects is promising. Further research is needed to investigate the efficacy of fluconazole as a denture cleansing agent and to determine appropriate dosage and treatment regimen. In the present study, fluconazole was the least effective at inhibiting *C. albicans* among all interventions. Sohnle and Hahn found that fluconazole was only significantly effective when treating *C. albicans* in mice for longer periods of time, for instance 4 weeks, 24 because fluconazole needs time for internalization into the nucleus to exert its effects and the mode of fungal inhibition of fluconazole is fungistatic not fungicidal. In the short time settings applied in the present study, 220-µM Ery + 1% TiO _2_ + BL showed superior anticandidal efficacy than fluconazole. Moreover, *Candida* has been shown to develop the ability to efflux azole drugs resulting in resistance, while Ery-mediated PDT develops no such resistance. 25 A systematic review and meta-analysis of anticandidal PDT found that indocyanine green, methylene blue, and hematoporphyrin have been used in clinical trials to treat *Candida* -related denture stomatitis. 26 Among these regimens, 1 mg/mL (∼1,320 µM) of indocyanine green in the presence of 810-nm diode at 56 J/cm ^2^ could effectively reduce *C. albicans* . 27 However, this high concentration was shown to reduce the viability of normal human cells. 28

Our findings concur with the report by Teerakapong et al 14 in which Ery at 220 µM + blue LED could inhibit *C. albicans* . When we compared the *C. albicans* inhibiting efficacy of 220-µM Ery + 1% TiO _2_ + BL with nystatin at 3 and 6 hours, the Ery group was slightly less effective. Nystatin mainly acts by binding ergosterol directly, leading to early, massive cell membrane damage and the leakage of intracellular ions. 29 Falah-Tafti et al showed nystatin was more effective than the systemic antifungal agent fluconazole at *C. albicans* inhibition in tissue conditioner, 30 which correlates with our findings in the present study. The inferior effect of PDT seen in this study might be due to the application of an inadequate energy density, based on the findings from a recent study that used 75 J/cm ^2^ to suppress *C. albicans* . 15 Additionally, we postulate that the relatively small amount of PDT-induced reactive oxygen species (ROS) produced by our reaction could not deeply penetrate the biofilm internal structure—the average thickness of a *C. albicans* biofilm is ∼15 μm. 31 Thus, the addition of another enhancer or using higher energy density may improve Ery + nano-TiO _2_ + BL PDT to be a novel effective alternative for treatment of denture stomatitis for intraoral use or as a denture cleanser.

The efficacy of 220-µM Ery + 1% TiO _2_ + BL *C. albicans* inhibition tended to increase with time, which is in concordance with the study of Teerakapong et al. 14 Nystatin showed a similar increase in anticandidal activity over time in our study ( *p* < 0.05, all times tested). This is in agreement with Taweechaisupapong et al who found that the efficacy of nystatin against *C. dubliniensis* increased up to 24 hours. 32 This may be explained by the fact that nystatin's chemical structure is quite large; thus, it can adhere to *C. albicans* cell walls for long periods before degradation. The amount of ROS formed in the present study was quite low and, with the short half-life of ROS, a prolonged reaction could not be achieved. Therefore, comparing the efficacy of nystatin and our PDT regimen using a higher light energy density and over a longer time to generate more ROS would be worthy of investigation.

The comparison between 220-µM Ery + 1% TiO _2_ + BL and Polident showed no difference in the efficacy of inhibiting *C. albicans* at 6 hours. However, several studies have found that denture soaking agents can cause denture surface roughness, color changes, 5 6 and allergy. 33 Polident reduces or prevents the adhesion of *C. albicans* to dentures 4 in addition to the antimicrobial action of H _2_ O _2_ . In contrast, 220-µM Ery + 1% TiO _2_ creates mainly singlet oxygen, which damages cells via physicochemical attack, but at a lesser reactive strength than H _2_ O _2_ . Polident cannot be directly used in the oral cavity due to its irritative and corrosive effects on the oral mucosa, but 220-µM Ery + 1% TiO _2_ has no such adverse effect 34 ; therefore, 220-µM Ery + 1% TiO _2_ might have advantages because it can be used directly on dentures as well as in intraoral sites. The efficacy of Polident tended to increase over time in the present study and a previous study showed significant increases in efficacy up to 16 hours ( *p* < 0.001). 35 However, this time course is impractical because denture wearers cannot immerse their dentures for such a long period. In contrast to discoloration and surface roughness possibly induced by Polident, 5 6 Ery resulted in negligible effect on denture discoloration.

When Ery is irradiated by BL, it generates singlet oxygen and free radicals 36 and it is the singlet oxygen that appears to be the most important molecule for the inhibition of microbes by Ery. 37 Singlet oxygen is very reactive toward biological tissue and will quickly react with unsaturated organic compounds and fatty acids. The plasma, mitochondrial, and nuclear membranes are rapidly destroyed by singlet oxygen. Enzymes and cellular proteins become crosslinked and may become bound to and inactivate DNA and RNA leading to apoptosis. 38 Teerakapong et al 14 previously used similar photosensitizers but with green LED and reported similar findings. However, the present study achieved a lower *Candida* inhibitory effect. This can be explained by the fact that Ery has peak absorption in the green light region with peak absorption at 530 nm. 39 Although using dental BL is practical, this may not generate adequate amounts of reactive oxygen species to kill *Candida* . Recently, Gonçalves et al used 100-μM Ery irradiated with 523 mW/cm ^2^ to yield an energy density of 63.8 J/cm ^2^ to generate a large amount of ROS that could reduce 6log _10_ CFU/mL of *C. albicans* in planktonic form. 21 This high energy density would probably induce damage to normal human intraoral cells. Thus, further investigations using energy densities in the range of 20 to 50 J/cm ^2^ and human cell viability tests are necessary to optimize the clinical application of Ery-TiO _2_ + BL-based antimicrobial PDT.

Nano-titanium dioxide has been used with photosensitizers such as Ery 14 and curcuminoid. 15 Nano-titanium, which is a photocatalyst, can increase the *C. albicans* inhibiting efficiency of Ery/curcuminoid by increasing the number of ROS while reducing the irradiation time. 14 15 Teerakapong et al compared the efficiency of Ery to inhibit *C. albicans* when used with or without 1% TiO _2_ . The presence of 1% TiO _2_ (w/w) + BL had more effect on inhibiting *C. albicans* . 14 Photocatalytic degradation of Ery was higher when used with nano-TiO _2_ . The dye molecules are converted to the triplet state via intersystem crossing system (ISC). The TiO _2_ semiconductor also absorbs light and as a result an electron–hole pair is generated, where an electron jumps from valence band to conduction band leaving behind a hole in the valence band. This electron will be abstracted by oxygen molecules (dissolved oxygen) generating superoxide anion radical (O2 ^−•^ ), which reacts with cell membrane. 40 Interestingly, chitin content can enhance the photocatalytic properties of nano-TiO _2_ 41 suggesting that nano-TiO _2_ impacts the cell wall and decreases the antioxidant molecule leading to mitochondria dysfunction and fragmentation.

The TEM images showed black NP with diameters less than 25 nm distributed in the cytoplasm, submembrane, and nuclear envelope of *C. albicans* . This result is in accordance with a previous study that showed TiO _2_ NP could enter *P. pastoris* cells and distribute in the cytoplasm, submembrane, nuclear envelop, and mitochondria. 42 In addition, TEM images indicated an increase in cell wall thickness, changes to cell shape, and cell wall and cell membrane damage over 1, 3, and 6 hours. Lara et al reported that treatment of *C. albicans* with silver NP enlarged the cell wall width via membrane permeabilization. 43 The present study is in line with a previous study that demonstrated that Ery-mediated PDT could destroy a part of the cell wall named blastoconidia. 36

This study was an *in vitro* study incorporating only a single species of *C. albicans* and, as such, may not mimic the clinical situation where multiple microbial species are orchestrating to enhance their tolerance. Similarly, although they are not expected to affect cell viability on their own, the effects of light only as well as photosensitizers only (nonirradiation group) on *C. albicans* were not included and future experiments should include a LIVE/DEAD assay to confirm the viability of the cells.

## Conclusion

At 1 hour, 220-µM Ery + 1% (w/w) TiO _2_ + BL was as effective as nystatin treatment for the inhibition of *C. albicans* . The anticandidal efficiency of 220-µM Ery + 1% (w/w) TiO _2_ + BL, Polident, and fluconazole were comparable within 6 hours. Transmission electron microscopy analysis revealed that the application of 220 µM Ery + 1% TiO _2_ + BL resulted in significant damage to both cell walls and cell membranes of *C. albicans* , including thickening of the cell walls and alteration of cell shape.

## Highlight

- Ery + TiO _2_ + BL could inhibit *C. albicans* biofilm on acrylic resin.
- At 1 hour, Ery + TiO _2_ + BL effectively decreased *C. albicans* equal to nystatin.
- 220 μM of Ery + TiO _2_ + BL caused *C. albicans* cell damage via cell wall rupture.
